# Prehospital damage-control amputation and packed red blood cell transfusion for severe bilateral lower-limb crush injury in a remote setting: a case report

**DOI:** 10.1093/jscr/rjag983

**Published:** 2026-10-27

**Authors:** Jianhua Li, Mingwei Ma, Qin Liu, Wei Tian, Jiangquan Li, Cheng Zhang

**Affiliations:** Department of Critical Care Medicine, Pengshui Miao and Tujia Autonomous County People's Hospital, No. 50, Wenmiao Street, Hanjia Subdistrict, Pengshui County, Chongqing 409600, China; Department of Critical Care Medicine, Pengshui Miao and Tujia Autonomous County People's Hospital, No. 50, Wenmiao Street, Hanjia Subdistrict, Pengshui County, Chongqing 409600, China; Department of Critical Care Medicine, Pengshui Miao and Tujia Autonomous County People's Hospital, No. 50, Wenmiao Street, Hanjia Subdistrict, Pengshui County, Chongqing 409600, China; Department of Critical Care Medicine, Pengshui Miao and Tujia Autonomous County People's Hospital, No. 50, Wenmiao Street, Hanjia Subdistrict, Pengshui County, Chongqing 409600, China; Department of Critical Care Medicine, Pengshui Miao and Tujia Autonomous County People's Hospital, No. 50, Wenmiao Street, Hanjia Subdistrict, Pengshui County, Chongqing 409600, China; Department of Cardiothoracic Surgery Intensive Care Unit, The First Affiliated Hospital of Chongqing Medical University, No. 1, Youyi Road, Yuanjiagang, Yuzhong District, Chongqing 400016, China

**Keywords:** amputation, case report, crush injury, damage control resuscitation, prehospital packed red blood cell transfusion, trauma

## Abstract

Prehospital damage-control amputation in civilian emergency systems is rare, especially combined with prehospital packed red blood cell transfusion. We report a 30-year-old man with bilateral lower-limb crush injury after entrapment in an industrial roller machine in a remote area. Despite initially preserved blood pressure, he developed marked hemodynamic deterioration and arrived at the hospital with a blood pressure of 74/49 mmHg. Both limbs were non-salvageable. Prehospital bilateral amputation with vascular ligation was performed on scene, followed by transfusion of one local unit of packed red blood cells (~200 ml). He survived after stump revision, intensive care, and rehabilitation. This case describes the sequential use of prehospital hemorrhage control and packed red blood cell transfusion during the same prehospital episode in a patient with a life-threatening entrapment injury and delayed transport.

## Introduction

Severe lower-extremity crush injuries in remote settings may require prehospital hemorrhage-control interventions before definitive care [1, 2]. Prehospital damage-control amputation is rare in civilian systems, with one specialist team reporting nine cases over 25 years [3], and its sequential use with prehospital packed red blood cell transfusion is infrequently described. We report the decision-making and management of bilateral prehospital damage-control amputation followed by packed red blood cell transfusion during the same prehospital episode. This case is reported in accordance with the SCARE 2023 guideline [4].

## Case report

At 09:55, a previously healthy 30-year-old man became entrapped by both legs in an industrial double-roller machine in a remote mountainous area. The township health-center team arrived at 10:36, applied tourniquets, established intravenous access, and started fluid resuscitation. Unable to manage the complex entrapment, the local team requested county-hospital specialist support; the trauma team arrived via mountainous roads at 13:18, 2 h and 42 min later. He remained conscious but was pale and diaphoretic; blood pressure 112/65 mmHg, heart rate 103 beats/min, and shock index 0.92 suggested evolving circulatory compromise. Serial vital signs and interventions are summarized in Table 1.

**Table 1:** Timeline of key prehospital and early in-hospital events.

Time	Event	Key findings and interventions
09:55	Bilateral limbs entrapped	Industrial machinery entrapment involving both lower limbs.
10:36	Primary team arrived	BP 133/95 mmHg; HR 113 beats/min; RR 18 breaths/min; GCS 15. Tourniquets applied; intravenous fluids initiated.
13:18	Trauma team arrived	BP 112/65 mmHg; HR 103 beats/min; RR 17 breaths/min; GCS 15.
13:40	Extrication completed	BP 111/72 mmHg; HR 100 beats/min; RR 15 breaths/min; GCS 15. Both limbs deemed non-salvageable before extrication.
13:43–14:03	Field amputation	Proximal vascular control and bilateral amputation; procedural blood loss ~300 ml. At completion: BP 112/75 mmHg; HR 98 beats/min; RR 16 breaths/min; GCS 15.
14:20	PRBC transfusion initiated	One local unit of uncrossmatched O Rh-positive PRBCs (~200 ml) initiated 4 h 25 min after injury. BP 112/65 mmHg; HR 108 beats/min; RR 16 breaths/min; GCS 15.
14:35	Transport initiated	BP 102/65 mmHg; HR 104 beats/min; RR 16 breaths/min; GCS 15.
14:50	PRBC transfusion completed	BP 112/64 mmHg; HR 98 beats/min; RR 17 breaths/min; GCS 15. No additional vital signs were documented before hospital arrival.
15:40	Hospital arrival	BP 74/49 mmHg; HR 98 beats/min; RR 16 breaths/min; GCS 14; SpO₂ 98%; temperature 36.5 °C. This was the lowest documented BP.
16:12	Stump revision and NPWT	Stump revision, debridement, definitive closure, and NPWT.
Postoperative Day 2	Extubation	Successfully extubated.

Prehospital fluids: from 10:36 to 13:18, 1000 ml NS and 500 ml HES; from 13:18 to 15:40, an additional 1750 ml NS and 1000 ml HES (total: 2750 ml NS and 1500 ml HES). Blood products: one local unit of PRBCs (~200 ml) prehospital; an additional 9.5 units of PRBCs (~1900 ml) and 1400 ml FFP in hospital. The estimated 300 ml blood loss refers only to field amputation; earlier blood loss could not be quantified.

BP, blood pressure; FFP, fresh frozen plasma; GCS, Glasgow Coma Scale; HES, hydroxyethyl starch; HR, heart rate; NS, normal saline; PRBCs, packed red blood cells; RR, respiratory rate; SpO₂, peripheral oxygen saturation.

Both limbs showed near-complete disruption distal to the proximal-to-mid-thigh level, with bilateral comminuted femoral, tibial, and fibular fractures, extensive muscle and tendon destruction, femoral vessels and major nerves transected or extensively destroyed, absent distal perfusion, and contamination by soil, straw, and machine oil. They were deemed non-salvageable before extrication. Proximal tissue destruction prevented effective tourniquet control, while machine compression was considered to provide partial tamponade. After complete release at 13:40, active bleeding became evident. Given the non-salvageable injuries, ineffective tourniquets, and delayed transport, immediate transfer for further limb assessment was not pursued; bilateral mid-thigh damage-control amputation began at 13:43 for hemorrhage control.

The patient gave verbal procedural consent; family members were absent and provided written confirmation after admission. Telephone authorization from the hospital vice president overseeing the trauma center was clinical, not research ethics, approval. Under combined spinal-epidural anesthesia, bilateral mid-thigh damage-control amputations with proximal vascular ligation were completed in ~20 min. Records lacked details on instruments, tourniquet and vessel-ligation management, bone division, stump treatment, aseptic measures, and anesthetic or analgesic agents and doses; intravenous analgesia without sedation and monitoring of blood pressure, heart rate, and oxygen saturation were documented. No immediate procedural or anesthetic complications occurred. Field-amputation blood loss was ~300 ml; earlier loss was unquantifiable. At 14:20, 4 h and 25 min after injury, one local unit of uncrossmatched O Rh-positive packed red blood cells (~200 ml), supplied by the hospital blood bank via cold chain, was started for active bleeding and evolving circulatory compromise. Prehospital fluids totaled 2750 ml normal saline and 1500 ml hydroxyethyl starch (Table 1); no transfusion reaction occurred, and tranexamic acid was not given. Written consent for publication and clinical photographs was subsequently obtained.

At 15:40, he arrived with blood pressure 74/49 mmHg, heart rate 98 beats/min, Glasgow Coma Scale score 14, and shock index 1.32; this was the lowest documented pressure. Admission laboratories showed hemoglobin 86 g/l, lactate 3.8 mmol/l, potassium 5.3 mmol/l, creatine kinase 1020 U/l, creatinine 44 μmol/l, prothrombin time 17.12 s, and fibrinogen 0.99 g/l. Injury Severity Score was 25. No additional injuries were documented; bedside pelvic radiography showed no injury, although CT and ultrasonography were not performed. Surgery confirmed bilateral limb loss distal to the proximal-to-middle thighs with irregular, exposed-bone, heavily contaminated stumps; revision, debridement, definitive closure, and negative-pressure wound therapy (NPWT) were completed in one operation. No further surgery was required, and NPWT was removed after 7 days. Final levels remained bilateral proximal-to-middle transfemoral amputations, without stump necrosis, infection, or recurrent bleeding. Intensive care included ventilation, antibiotics, and an additional 9.5 local units of packed red blood cells (~1900 ml) and 1400 ml of fresh frozen plasma.

He was extubated on postoperative Day 2 and transferred to the ward on intensive care unit Day 4. No transfusion reaction or acute kidney injury occurred; renal function and urine output remained normal. After ~2 weeks, he was discharged to rehabilitation with healed stumps, which remained uninfected at 1 month. At 3 months, he used temporary bilateral prostheses for gait training. He could stand but not walk independently, requiring a walker, crutches, or assistance; he remained independent in basic activities of daily living and wheelchair transfers and reported mild phantom limb pain.

Table 1 summarizes the clinical timeline; Figs 1 and 2 show prehospital management and 3-month follow-up, respectively.

**Figure 1:**
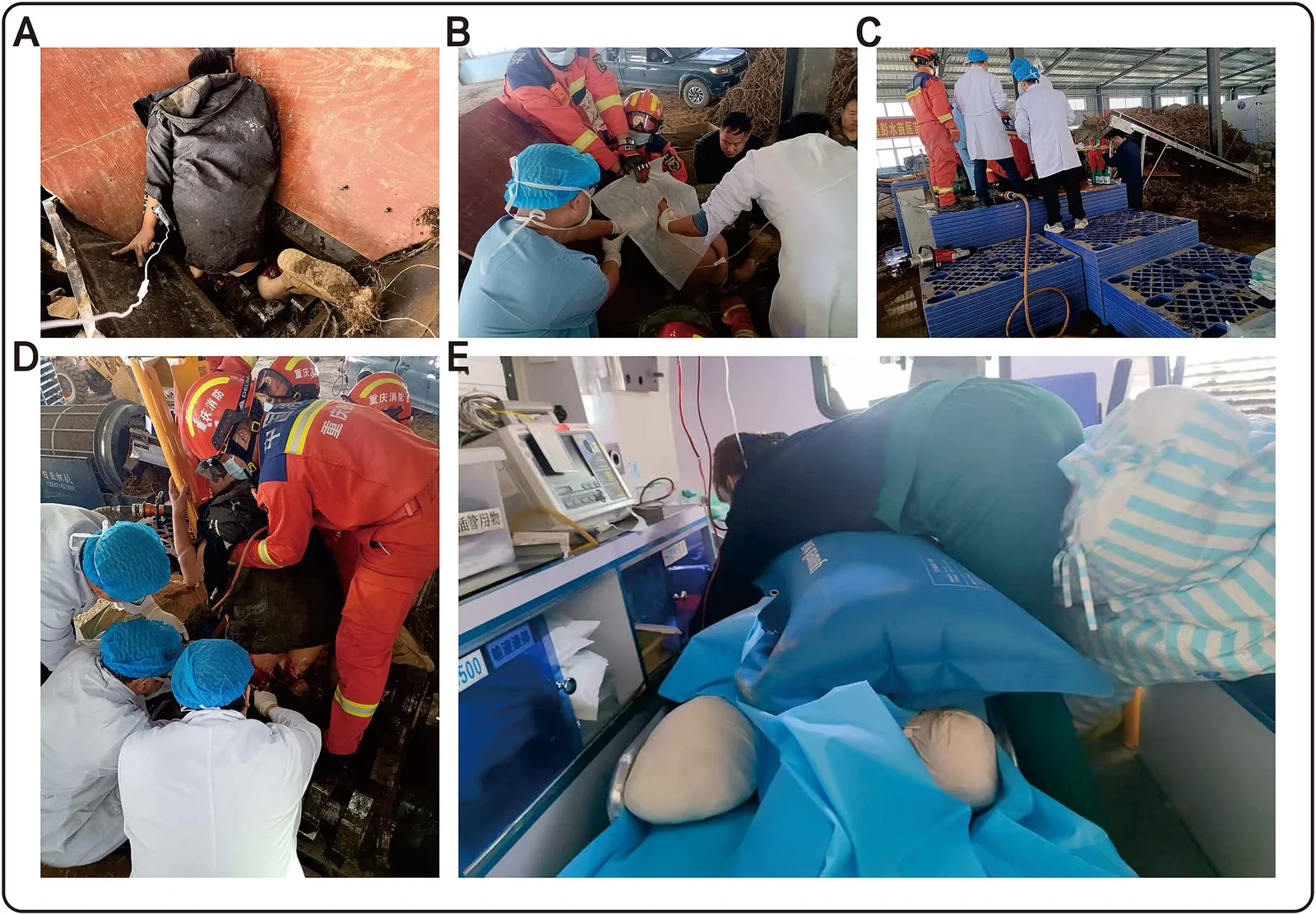
Prehospital management process. (A) Both lower limbs entrapped in an industrial mechanical device; (B) prehospital medical team performing initial assessment; (C) mechanical extrication; (D) field amputation for hemorrhage control; (E) transport under continuous resuscitation and monitoring.

**Figure 2:**
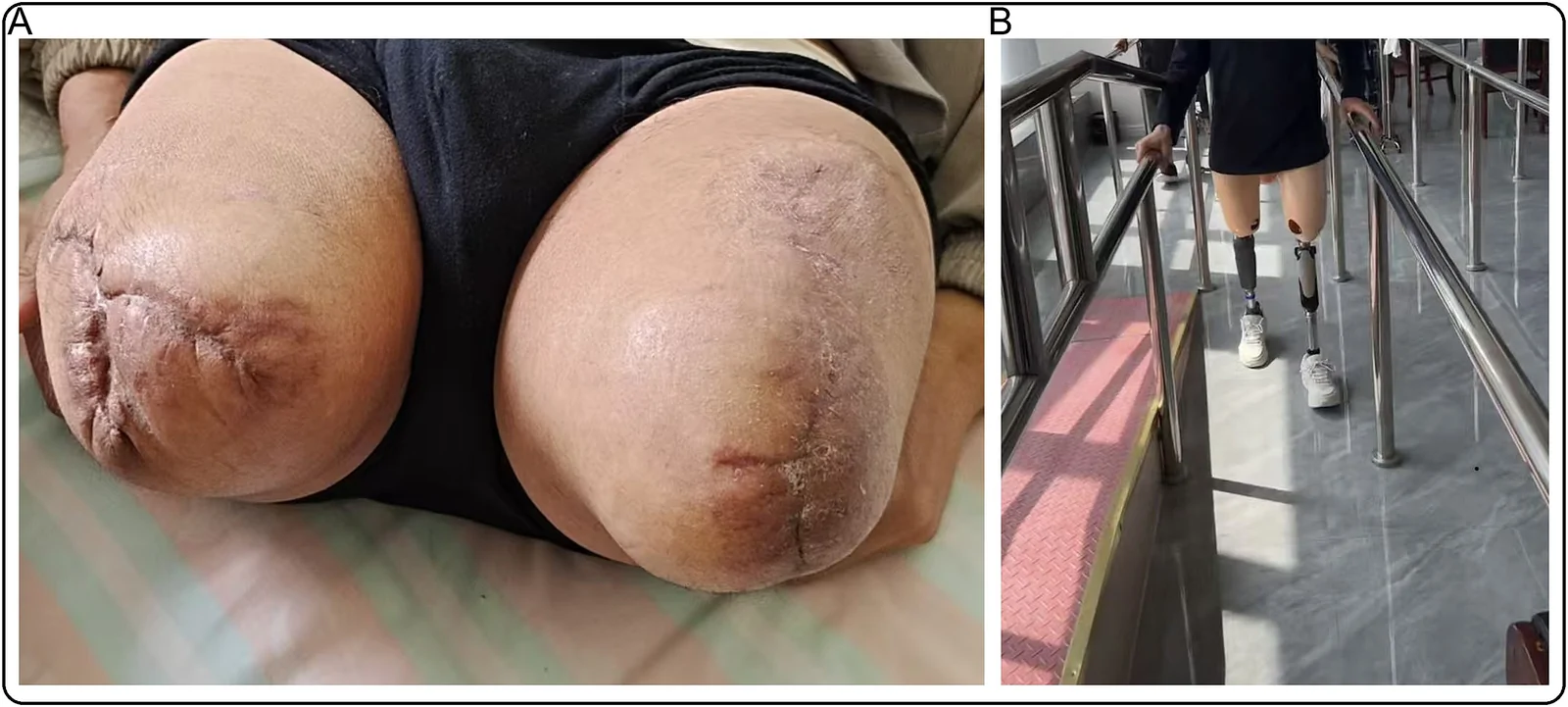
Three-month postoperative follow-up. (A) Bilateral transfemoral residual limbs with healed surgical scars. (B) Assisted gait training with temporary bilateral prostheses using parallel bars.

## Discussion

Prehospital damage-control amputation is rare but may be required for non-salvageable limbs when hemorrhage cannot otherwise be controlled [3, 5]. Contemporary guidance identifies field amputation, regional anesthesia, blood-product transfusion, and emergency medical services physician response among measures used for complex entrapment [5]. In this case, machine compression provided partial tamponade; bleeding after decompression and ineffective tourniquets prompted amputation before prolonged transport. Although blood pressure remained preserved during the procedure, it fell to 74/49 mmHg by hospital arrival; thus, local hemorrhage control did not equate to hemodynamic stabilization.

Evidence for prehospital transfusion remains uncertain. Resuscitation with Pre-Hospital Blood Products (RePHILL) did not demonstrate superiority of packed red blood cells plus lyophilized plasma over saline for its composite outcome of mortality and impaired lactate clearance, and European guidance makes no recommendation for or against prehospital blood products [6, 7]. Here, one unit of packed red blood cells served as a temporizing measure between field hemorrhage control and hospital arrival; this case cannot establish benefit or transport safety.

Generalizability is limited by the single-case design, escalation from a township health-center team to a county-level trauma team, travel over mountainous roads, and technically complex extrication. Detailed procedural records and comprehensive trauma imaging were unavailable, and functional follow-up ended at 3 months. This report therefore documents sequential field hemorrhage control and transfusion within one prehospital episode without supporting broad recommendations.

## Data Availability

All data are included in this article. Additional information is available from the corresponding author upon reasonable request, subject to patient privacy.
